# Metabolic profiling in children and young adults with autosomal dominant polycystic kidney disease

**DOI:** 10.1038/s41598-021-84609-8

**Published:** 2021-03-23

**Authors:** Madhurima M. Baliga, Jost Klawitter, Uwe Christians, Katharina Hopp, Michel Chonchol, Berenice Y. Gitomer, Melissa A. Cadnapaphornchai, Jelena Klawitter

**Affiliations:** 1 University of Colorado Denver, School of Medicine, Aurora, CO USA; 2 Deparment of Anesthesiology, University of Colorado Denver Anschutz Medical Campus, 12705 E Montview Blvd., Bioscience 2, Suite 200, Aurora, CO 80045-7109 USA; 3 Division of Renal Diseases and Hypertension, University of Colorado School of Medicine, Aurora, CO USA; 4 Presbyterian/St. Luke’s Medical Center, Denver, CO USA

**Keywords:** Biomarkers, Nephrology

## Abstract

Autosomal dominant polycystic kidney disease (ADPKD) is the most commonly inherited kidney disease. Although children with ADPKD show normal renal function, cyst development is already occurring. In this study, we aimed to identify markers and associated molecular pathways of disease progression in children and young adults with ADPKD. Plasma samples were collected during a 3-year randomized, double-blind, placebo-controlled, phase III clinical trial that was designed to test the efficacy of pravastatin on slowing down ADPKD progression in pediatric patients. Samples from 58 patients were available at baseline and at the 3-year endpoint of the study, respectively. Furthermore, plasma samples from 98 healthy children were used as controls. Metabolomic analysis was performed using liquid chromatography-tandem mass spectrometry and differences in metabolic profiles over time and within study groups were evaluated. While pravastatin therapy led to a decrease in a percent change of total kidney volume (HtTKV) in ADPKD patients, it had minimal effects on metabolite changes. Oxidative stress, endothelial dysfunction, inflammation and immune response were the most affected signaling pathways that distinguished healthy from diseased children. Pathway analysis revealed that metabolites in the arginine metabolism (urea and nitric oxide cycles), asparagine and glutamine metabolism, in the methylation cycle and kynurenine pathway were significantly changed between healthy and children with ADPDK and continued to diverge from the control levels while the disease progressed. Detected metabolite changes were primarily governed by disease progression, and less by pravastatin treatment. Identified metabolic pathways, from arginine and asparagine to kynurenine metabolism could present therapeutic targets and should be further investigated for potential to treat ADPKD progression at an early stage.

## Introduction

Autosomal dominant polycystic kidney disease (ADPKD) is the most commonly inherited kidney disease, affecting approximately 1:400 to 1:1000 live births. The disease is characterized by cysts in the kidneys as well as extrarenal manifestations (e.g. hepatic cysts, mitral valve prolapse, berry aneurysms) and accounts for 5% of the end stage renal disease (ESRD) population^1,2^. In ADPKD patients, cyst growth and accumulation are thought to compress renal vasculature and activate the renin–angiotensin–aldosterone system (RAAS)^3^. RAAS activation along with the upregulation of vasopressin receptors in these patients leads to early hypertension^4–7^. In children with ADPKD, early onset of hypertension has been linked to rapid decline in estimated glomerular filtration rate (eGFR) and early-onset ESRD^8,9^.

In addition to early hypertension, factors including *PKD1* versus *PKD2* mutation, gender, early and frequent gross hematuria as well as changes in total kidney volume (TKV), glomerular filtration rate (GFR), and renal blood flow are linked to early adverse outcomes in ADPKD^10^. Given that treatments for ADPKD are limited and that a subset of patients may benefit from early intervention, we conducted a randomized clinical trial to test the efficacy of pravastatin on slowing disease progression in children and young adults with ADPKD. The results showed that the percent change in height-corrected TKV (HtTKV) over the 3-year period, following adjustment for age, sex, and hypertension status, was significantly decreased with pravastatin therapy^11,12^.

Furthermore, in addition to limited therapy options, there is also still a lack of reliable prognostic or predictive biomarkers in children and adults with ADPKD.

Metabolomics, the study of the small-molecule intermediates of cellular metabolism, has been useful in eliciting metabolic pathways that underlie disease processes as well as in identifying biomarkers of disease progression^13^. Whereas several metabolomics studies were performed in patients with chronic kidney disease (CKD) and some in adult patients with ADPKD^14–18^, only a handful of studies have looked at biomarkers in pediatric patients with ADPKD^19^. Our previous work focused on identifying changes in biomarkers of inflammation and oxidative stress, both processes that underly the development and progression of ADPKD. We found that pravastatin therapy diminished the increase of cyclooxygenase- (COX) and lipoxygenase-derived (LOX) pro-inflammatory and oxidative stress markers in plasma of children and young adults with ADPKD^19^. Thus, in the present study, we utilized the same plasma samples collected at baseline and at the 3-year endpoint of the above referenced pediatric clinical trial for targeted metabolomics analysis with the aim to identify additional markers, metabolic pathways and mechanisms that are associated with ADPKD progression^11,12,19^.

## Materials and methods

### Trial design

For this study, we used plasma samples collected during the Pravastatin ADPKD Pediatric Clinical Trial^12^. Briefly, a 3-year randomized, double-blind, placebo-controlled phase 3 clinical trial was designed to investigate the effect of pravastatin therapy on change in the combined endpoints of HtTKV, left ventricular mass index (LVMI), and urinary microalbumin excretion (UAE) in pediatric ADPKD patients^11,12^. Patients were seen for a baseline visit during which a history and physical exam were undertaken, abdominal and cardiac MRI scans were performed, and fasting blood work (at least 10 h) was collected. All patients were placed on the angiotensin-converting enzyme (ACE) inhibitor, lisinopril. Patients were seen again at 36 months (i.e. 3-year endpoint of the study) at which time the tests mentioned above were repeated^11,12^.

This study was registered and approved (NCT00456365, April 4, 2007) by the Colorado Multiple Institutional Review Board (COMIRB). Written informed assent/consent was obtained. Study conduct followed the principles of good clinical practices as monitored by the local IRB and adhered to the principles as set forth by the Declaration of Helsinki and its amendments.

### Healthy subjects

Our control group consisted of plasma samples collected from 98 healthy children undergoing minor dental and ENT surgical procedures (patients fasted for at least 8 h prior to surgery). Sample collection was approved by COMIRB and occurred at the same clinical site (Children’s Hospital Colorado). Upon plasma generation, all samples (from diseased and healthy subjects) were stored at − 80°C.

### Targeted metabolomics

Collected plasma samples were extracted according to a published protocol^20^. Briefly, samples were centrifuged and mixed with methanol to create an 80% (volume/volume) methanol solution and were incubated overnight at − 80°C to allow for protein precipitation. Following incubation, samples were centrifuged, and supernatants were dried in a SpeedVac concentrator (Savant, ThermoFisher, Waltham, MA). Samples were reconstituted with 20µL water/methanol (80:20, volume/ volume). Selected multiple reaction monitoring (sMRM) of 184 metabolites using a positive/negative ion-switching high-performance liquid chromatography-tandem mass spectrometry (5500 QTRAP HPLC–MS/MS^21^) was used for analysis (please refer to Supplementary Materials for more detail).

Once the data were acquired, MultiQuant (v2.1.1., Sciex, Foster City, CA) software was used for data processing of 184 unique metabolites in plasma. For between-sample normalization, the intensity values for each sample were summed up, and the median value of the sums across all samples were determined. Tune and quality control samples were evenly distributed during the batches. The intensity values of each sample were then scaled such that the sum of the scaled intensities equaled the median value of all samples. Normalized intensity values were then log_2_ transformed to reduce the influence of extreme values and to meet the homogeneity of variance assumption.

### Statistical analysis

MetaboAnalyst 4.0 was used for statistical analysis of metabolomics data^22^. Changes in metabolites between healthy and diseased as well as between placebo and pravastatin treated ADPKD patients was performed by utilizing Principal Component Analysis (PCA). Relative peak intensities were initially log transformed and then autoscaled (mean centered and divided by the square root of the SD of each variable). Pathway analysis was performed using the pathway analysis tool in MetaboAnalyst 4.0 (University of Alberta, Canada). This tool uses both pathway enrichment analysis through the R‐package GlobalTest based on compound concentration values as well as pathway topological analysis accounting for the impact of individual measured metabolites within the pathway. The goal of assessing pathway impact is to account for pathway structure and the intuitive concept that central or nodal positions in a pathway will have a greater impact than marginal or isolated positions. Total or maximal importance for each pathway is designated as 1, whereas the importance of measured metabolites to that pathway is designated as the cumulative percentage from matched metabolite nodes. Stepwise linear regression analysis and correlation analysis was performed to identify relationships between the individual metabolites and HtTKV at baseline as well as percent change in metabolites and the percent change in HtTKV (36 months versus 0 months), after adjustment for age, gender, and treatment group using SPSS 27.0 (IBM, Armonk, NY). Percent change was calculated by normalizing change over time (Δ36 months – baseline) to the corresponding baseline value. Bonferroni correction was applied to correct for multiple testing.

Finally, sensitivity and specificity of biomarkers was analyzed in MetaboAnalyst using multivariate Receiver Operator Characteristic (ROC) curve analysis, with a P < 0.05 considered significant.

## Results

### Patient characteristics

From 110 participants enrolled in the clinical trial, plasma samples from 78 patients were available at baseline, and samples from 58 patients were available at both baseline and 36-month time points (Tables 1, 2). All available samples were utilized for analysis. There were no significant changes in the renal function (eGFR) between the groups, either at the baseline or at the end of the trial (Tables 1, 2). HtTKV increased from 342 ± 213 ml/m (baseline) to 441 ± 267 ml/m (36 months) in placebo group patients^11,12^ (Tables 1, 2). Characteristics of the healthy subjects are summarized in Table 3. While we did not evaluate the renal function in the healthy children, it can be expected that their 24-h urine creatinine clearance [mL/min/1.73 m^2^] was within the established reference range of 85–145 (5–95 percentile in children above 2 years of age)^23,24^. In the vast majority of children and young adults with ADPKD, kidney function is within the normal range^25,26^ (Tables 1, 2).

**Table 1:** Characteristics of the patients at the initial visit (N = 58).

Characteristic	Placebo (N = 27)	Pravastatin (N = 31)	P Value
Age (year)	15 ± 4	16 ± 3	0.82
% Female	59	55	
**Race**			
% White	93	100	
% Black	7	0	
% Other	0	0	
Height (cm)	161 ± 15	167 ± 16	0.16
Weight (kg)	56 ± 20	67 ± 23	0.05
**Urine microalbumin excretion (mcg/min)**			
Mean	42 ± 96	19 ± 23	0.25
Median	15 [7, 23]	10 [7, 19]	
Left ventricular mass index (g/m^2^)	53 ± 11	55 ± 13	0.65
**Total kidney volume (ml)**			
Mean	565 ± 375	545 ± 250	0.81
Median	489 [322, 663]	493 [324, 668]	
**Total kidney volume corrected for height (ml/m)**			
Mean	342 ± 213	321 ± 136	0.67
Median	278 [196, 386]	287 [200, 372]	
**Serum creatinine (mg/dl)**			
Mean	0.6 ± 0.2	0.7 ± 0.2	0.31
Median	0.6 [ 0.5, 0.7]	0.7 [0.6, 0.8]	
24-h urine creatinine clearance (ml/min per 1.73 m^2^)	142 ± 34	140 ± 32	0.80
**BP (mmHg)**			
Systolic	119 ± 11	122 ± 11	0.42
Diastolic	72 ± 7	72 ± 7	0.97
**24-h urine protein (g/day)**			
Mean	0.16 ± 0.20	0.11 ± 0.05	0.25
Median	0.11 [ 0.09, 0.12]	0.10 [0.07, 0.14]	
Hematocrit (%)	39 ± 3	41 ± 3	**0.03**
**Cholesterol**			
HDL	49 ± 11	48 ± 13	0.78
LDL	94 ± 22	85 ± 23	0.14
Total	157 ± 26	145 ± 25	0.07

Samples from the same patients were available at the baseline and the 36-month time points (as reported in^12^). Values are the mean ± SD or median [25th percentile, 75th percentile]. P < 0.05 considered significant (bolded).

**Table 2:** Characteristics of the patients at the final visit (N = 58).

Characteristic	Placebo (N = 27)	Pravastatin (N = 31)	P Value
Age (year)	18 ± 4	19 ± 3	0.84
% Female	59	55	
**Race**			
% White	93	100	
% Black	7	0	
% Other	0	0	
Height (cm)	170 ± 11	172 ± 12	0.39
Weight (kg)	64 ± 18	75 ± 22	**0.04**
**Urine microalbumin excretion (mcg/min)**			
Mean	27 ± 43	18 ± 15	0.29
Median	13 [8, 23]	13 [10, 19]	
**Left ventricular mass index (g/m**^**2**^**)**			
Percent change for slow progressors (per 3 year, within patient, N = 13)	− 14.3 ± 7.3	− 15.5 ± 8.6	0.80
Percent change for fast progressors (per 3 year, within patient, N = 13)	48.5 ± 27.4	38.5 ± 27.1	0.53
**Total kidney volume (ml)**			
Mean	758 ± 473	680 ± 380	0.50
Median	610 [403, 965]	527 [422, 868]	
Percent change (per 3 year, within patient)	40 ± 27	26 ± 16	**0.03**
**Total kidney volume corrected for height (ml/m)**			
Mean	441 ± 267	388 ± 200	0.40
Median	342 [245, 542]	310 [250, 480]	
Percent change (per 3 year, within patient)	31 ± 21	22 ± 14	**0.04**
Percent change for slow progressors (per 3 year, within patient, N = 13)	7.8 ± 3.0	6.8 ± 4.1	0.62
Percent change for fast progressors (per 3 year, within patient, N = 13)	63.4 ± 10.8	42.3 ± 6.5	**0.003**
**Serum creatinine (mg/dl)**			
Mean	0.7 ± 0.1	0.7 ± 0.1	0.65
Median	0.7 [0.6, 0.8]	0.7 [0.7, 0.8]	
24-h urine creatinine clearance (ml/min per 1.73 m^2^)	124 ± 24	135 ± 25	0.10
**BP (mmHg)**			
Systolic	121 ± 9	122 ± 11	0.92
Diastolic	74 ± 8	73 ± 9	0.56
**24-h urine protein (g/day)**			
Mean	0.16 ± 0.11	0.13 ± 0.09	0.31
Median	0.14 [0.09, 0.19]	0.11 [0.06, 0.17]	
Hematocrit (%)	41 ± 3	42 ± 4	0.48
**Cholesterol**			
HDL	50 ± 12	47 ± 14	0.36
LDL	89 ± 28	73 ± 27	**0.04**
Total	160 ± 31	142 ± 36	0.05

Samples from the same patients were available at the baseline and the 36-month time points (as reported in^12^). Values are the mean ± SD or median [25th percentile, 75th percentile]. P < 0.05 considered significant (bolded).

**Table 3:** Characteristics of the healthy subjects (N = 98).

Characteristic	Healthy controls (N = 98)
Age (year)	12 ± 5
% Female	53
**Race**	
% White	90
% Black	2
% Other	6
% Unknown	2

### Consistency with parent clinical trial

Since only a subset of patients’ plasma samples were available at both baseline and 36-month time points, we aimed to confirm the consistency of findings in our reduced subset with the outcomes of the parent clinical trial. Our results showed a significant decline in the change of HtTKV over 3 years (Supplementary Figure 1, Table 2) and were consistent with the results of the parent trial^12^.

### Comparison with healthy children

**Figure 1:**
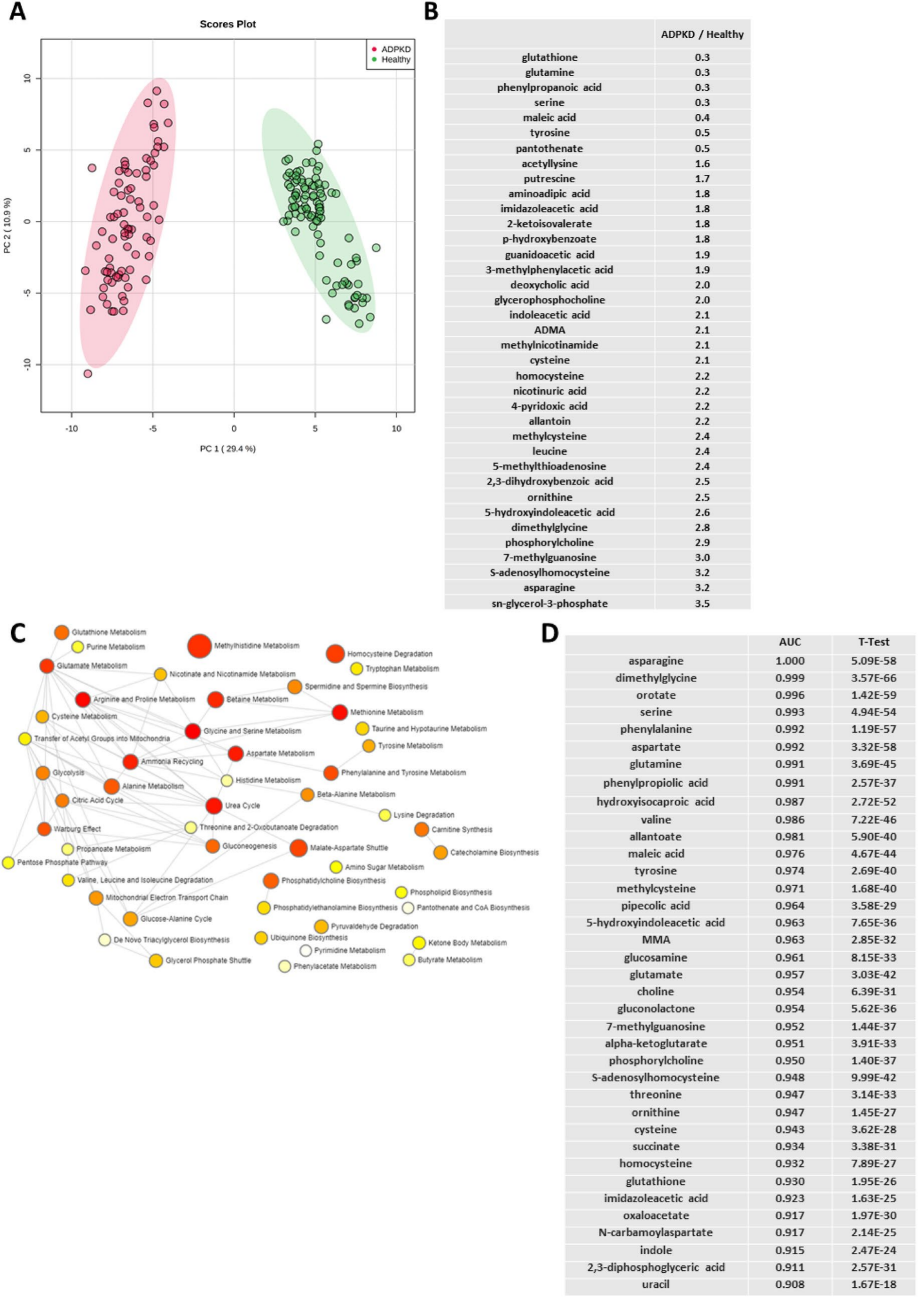
(**A**) Principal component analysis scores plot of healthy children (N = 98) versus children with ADPKD (N = 78) revealed a clear separation between the groups at baseline. Ellipses represent 95% confidence intervals for each individual group on the PCA plot. (**B**) Factor change in metabolite intensity between patients with ADPKD and healthy subjects (at baseline; presented only those with minimum of 50% change) and (**C**) pathway enrichment analysis of metabolites that were significantly different (after Bonferroni correction) between healthy children and children with ADPKD at baseline. The color and size of each dot were associated with the -log (*p*) value and pathway impact value, respectively, where a small p value and high pathway impact value indicate the pathway is greatly influenced (large red node). (**D**) Receiver operating curve (ROC) biomarker analysis of metabolites identified in ADPKD at baseline versus healthy subjects revealed thirty-seven metabolites with area under the curve (AUC) values of above 0.90.

As expected, we observed a clear separation between the baseline metabolic profiles of children with ADPKD and healthy children (Fig. 1A). Ninety-five metabolites were statistically significantly changed between the two groups (after Bonferroni correction), and thirty-nine of these had changed more than 50% between the groups (Fig. 1B). As aforementioned, kidney function did not change between the baseline and the end of the trial in either of the treated patient groups^12^. It should be mentioned that, while all patients were fasting for at least 8 h prior to sample collection, we did not monitor their prior dietary or water intake. In addition, healthy subjects were on average 4 years younger than the patients with ADPKD, and therefore ANCOVA was used to correct for age differences.

The pathway enrichment analysis performed on the above mentioned 95 compounds revealed methylhistidine metabolism, homocysteine degradation, malate-aspartate shuttle, betaine metabolism and urea cycle to be the most affected pathways and separators between the children with ADPKD and those without. Furthermore, amino acid metabolism (including glutamate, arginine and proline metabolism, alanine, cysteine, aspartate, serine and glycine metabolism); glucose metabolism (including glycolysis, citric acid cycle and gluconeogenesis) as well as fatty acid metabolism and oxidation (including phospholipid and triacylglycerol biosynthesis and transfer of acetyl groups into mitochondria) were identified as the main metabolic nodes connecting the metabolic pathways significantly different between the healthy and diseased children (Fig. 1C).

Biomarker analysis using receiver operating characteristic (ROC) curves revealed thirty-seven metabolites with area under the curve (AUC) values of above 0.90 and thus an excellent sensitivity potential for distinguishing healthy from children with ADPKD (Fig. 1D).

### Baseline cross-sectional metabolite analysis

**Figure 2:**
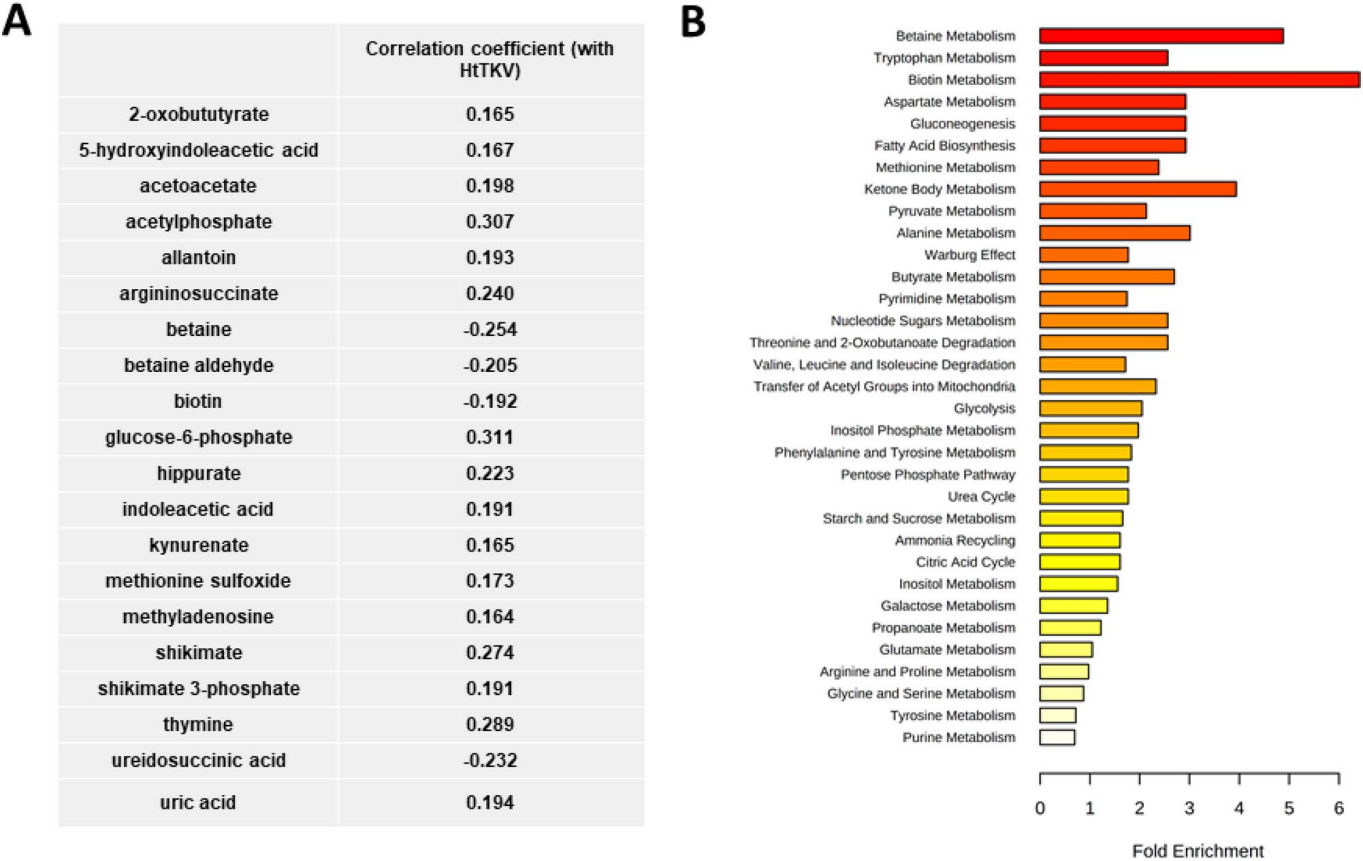
(**A**) Pearson correlation coefficients and (**B**) pathway enrichment analysis of plasma metabolites that significantly correlated with HtTKV at baseline in pediatric ADPKD patients (N = 78, after adjustment for age, gender and race).

PCA analysis showed that metabolic profiles of patients were, as expected, similar prior to randomization (Supplementary Figure 2, no significant features between groups). At baseline, twenty-one metabolites significantly correlated with the patients’ HtTKV, after adjustment for age, gender and race, and again, prior to any change in GFR (Fig. 2A). Several of the metabolites including allantoin, uric acid, 1-methyladenosine, indoleacetic acid, kynurenate, thymine and hippurate are known uremic toxins and have been shown to be associated with the progression of chronic kidney disease and worsening of renal function^14,27–30^. Allantoin, 5-hydroxyindoleacetic acid (5-HIAA) and indoleacetic acid were metabolites that we identified above as significantly different between healthy and children with ADPKD.

The identified metabolites belonged to the betaine metabolism, ketone body metabolism, gluconeogenesis and fatty acid biosynthesis, as well as aspartate and tryptophan metabolism pathways (Fig. 2B). Interestingly, most of the metabolites that associated with HtTKV in a stepwise linear regression analysis in ADPKD patients at baseline were also those we identified as being different between children and young adults with ADPKD at baseline and healthy children (Fig. 1C,D).

### Markers of disease progression

**Figure 3:**
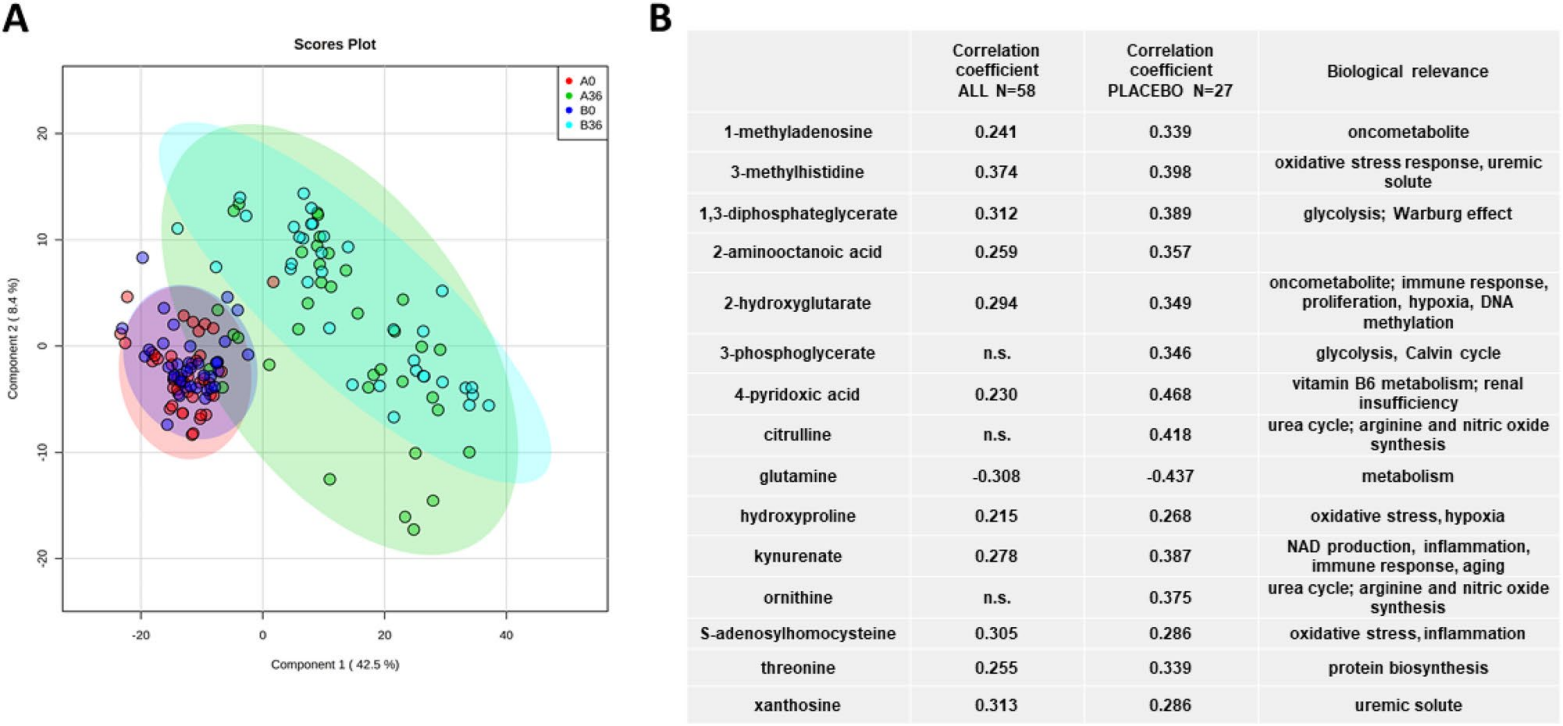
(**A**) Principal component analysis between pediatric ADPKD patients at the baseline (0 months) and after 36 months of treatment (group A = pravastatin, N = 31, group B = placebo, N = 27) and (**B**) Pearson correlation coefficients between the percent change HtTKV to a percent change in metabolites (significant after adjustment to age, gender, race (placebo, N = 27) as well as treatment group (both groups combined = ALL, N = 58). Percent change was calculated by normalizing the change over time (Δ(36–0) months) to the corresponding baseline. Ellipses represent 95% confidence intervals for each individual group on the PCA plot.

As the disease progressed, plasma metabolomic profiles of placebo and pravastatin treated ADPKD patients separated from their respective baselines (Fig. 3A). However, very little separation between the placebo and pravastatin groups was visible at 36 months, suggesting limited effect of statin treatment on the change in metabolite profiles identified by the presently used metabolomics assay (Fig. 3A). Furthermore, since patients started the study with different baseline values, we normalized the change in metabolites over time (Δ(36–0) months) to the corresponding baseline value (as percent change). Again, there was a lack of separation between placebo and pravastatin groups (data not shown).

To specifically address the question of which metabolites correlated with increasing HtTKV and thus worsening ADPKD disease, we performed stepwise linear regression and correlation analysis between percent change in metabolites to percent change in HtTKV (adjusted for age, gender and race, Fig. 3B). As previously shown, due to the lack of distinction between placebo and pravastatin groups at either timepoint, we decided to perform analysis on only the placebo group (N = 27) as well as on combination of both treatment groups and so to improve the statistical power for discovery of markers and pathways related to ADPKD progression (N = 58). Strong correlations, after adjustment for gender, age and race (and treatment group when both groups were considered) were observed between percent change in HtTKV and methylhistidine and methyladenosine, both markers shown to be elevated in patients with advanced chronic kidney disease^31^. Methyladenosine, ornithine and kynurenate were the three metabolites that were significantly associated with HtTKV at baseline (Fig. 2A) as well as with the change in HtTKV over the 3-year observation period (Fig. 3B).

Most importantly, we identified metabolites that were significantly different between healthy and children with ADPKD (at baseline, after Bonferroni correction) and that were also associated with disease progression (expressed as the change in HtTKV over 3 years, adjusted for age, gender, and race) while further differentiating from healthy levels (Table 4). Identified metabolites were associated with tryptophan metabolism, urea cycle, ammonia recycling, aspartate and glutamine, nicotinamide, methionine, arginine and proline, glycine and serine metabolism as well as the Warburg effect.

**Table 4:** Summary of metabolites that were significantly different between healthy children (N = 98) and children with ADPKD at baseline (N = 78) AND were significantly associated after linear regression analysis with either HtTKV at baseline (adjusted for age, gender and race, N = 78) or with a percent change in HtTKV over 3 years (adjusted for age, gender, treatment groups and race, N = 58).

Significantly different between healthy subjects and pediatric patients at the initiation of the trial AND	
Association with HtTKV at baseline	Association with percent change in HtTKV over 3 years
2-Oxobututyrate	1,3-Diphosphateglycerate
5-Hydroxyindoleacetic acid	2-Aminooctanoic acid
Acetoacetate	2-Hydroxyglutarate
Acetylphosphate	2-Ketohexanoic acid
Allantoin	4-Pyridoxic acid
Betaine	Glutamine
Glucose-6-phosphate	Hydroxyproline
Indoleacetic acid	Kynurenine
Kynurenine	Methyladenosine
Methionine sulfoxide	Ornithine
Methyladenosine	S-Adenosylhomocysteine
Ornithine	Xanthosine
Shikimate	
Shikimate 3-phosphate	
Thymine	

## Discussion

In ADPKD, it is clinically very difficult to identify patients at risk for rapid disease progression. The literature on biomarkers of disease progression is scarce in general and even more so in pediatric patients. Thus, identification of biomarkers and metabolic pathways at early stages of ADPKD, before secondary effects including the vicious cycle of oxidative stress and inflammation emerge, is of utmost clinical importance. The present study characterized plasma fingerprints of pediatric ADPKD patients with preserved kidney function. Targeted metabolomics profiling was performed with the following aims: (1) to identify the markers and pathways that differ between healthy children and those with ADPKD and (2) to identify if these or other markers and pathways associate with the progression of ADPKD over 36 months.

We could demonstrate that PCA-based classification of metabolic fingerprints allowed for a reliable discrimination between children with very early stage ADPKD and healthy children. Our data suggest the existence of a set of compounds and metabolic pathways sensitive to early stages of ADPKD. These included ninety-five metabolites involved in the metabolism of amino acids, carbohydrates, nucleotides, lipids, vitamins and cofactors as well as phase II-conjugation drug metabolism.

A large number of the identified metabolites are known uremic toxins including 1-methyladenosine, allantoin, asymmetric dimethylarginine (ADMA), dimethylglycine (DMG), guanidinoacetic acid (GAA), homocysteine (HCy), hypoxanthine, indolacetic acid (IAA) and 5-hydroxyindolecateic acid (5-HIAA), kynurenate, monomethylarginine (MMA), quinolinate, trimethylamine oxide (TMAO), and xanthine^30,32,33^. Their accumulation in plasma of ADPKD as compared to healthy children was evident even prior to the decline of GFR. From the ninety-five identified metabolites, thirty-seven showed potential to differentiate between the healthy and diseased pediatric populations as identified by ROC analysis.

### Arginine metabolism (urea and nitric oxide cycles) and methylation cycle

Children with ADPKD showed unchanged arginine levels but significantly higher levels of ornithine and the polyamine putrescine as well as a lower level of citrulline as compared to healthy children. The enzyme responsible for the conversion of arginine to ornithine is arginase (Arg), whose overexpression and increased activity leads to a reduced bioavailability of arginine for nitric oxide (NO) production and therefore to oxidative stress and endothelial dysfunction^34,35^. Furthermore, increases in arginase activity and accumulation of polyamines have been linked to cancer and dysfunction of the immune system^36,37^.

In terms of ADPKD, comparison of gene expression profiles in kidney tissues of *Pkd1*-deficient versus wild-type mice identified 204 genes that were differently expressed in late-stage polycystic kidneys including *Arg1*. Said results indicated that arginine metabolism was significantly activated in *Pkd1*-KO mice^38^. Arg1 was predominantly expressed in macrophages. Inhibition of Arg1 activity significantly retarded cyst growth and effectively lowered the proliferative indices in polycystic kidneys^38^. In vitro experiments revealed that macrophages with an upregulated Arg1 expression and increased polyamine synthesis stimulated cyst-lining epithelial cell (CLEC) proliferation^38^. Another study showed that the expression of ASS1, an enzyme that converts aspartate to argininosuccinate, which is needed for arginine production, is reduced in murine and human ADPKD, and that arginine depletion results in a dose-dependent compensatory increase in ASS1 levels as well as decreased cystogenesis in vitro and ex vivo with minimal toxicity to normal cells^16^. Change in citrulline and ornithine over 36 months positively corelated with the corresponding change in HTKV in lisinopril-treated placebo patients. Interestingly, after 36 months, both treatments, lisinopril alone and lisinopril plus pravastatin were able to reduce the ornithine/arginine-ratio, suggesting a decrease in arginase activity. The effects were stronger in the combination therapy group, similar to previous observations made in patients treated with ACE inhibitors with or without statins^35^. While arginine/ argininosuccinate and arginine/citrulline ratios remained unchanged over the 3 years of treatment, concentrations of eNOS inhibitors ADMA and SDMA declined, suggesting an improvement in eNOS activity with ACE inhibition.

### Glutamine and asparagine metabolism

**Figure 4:**
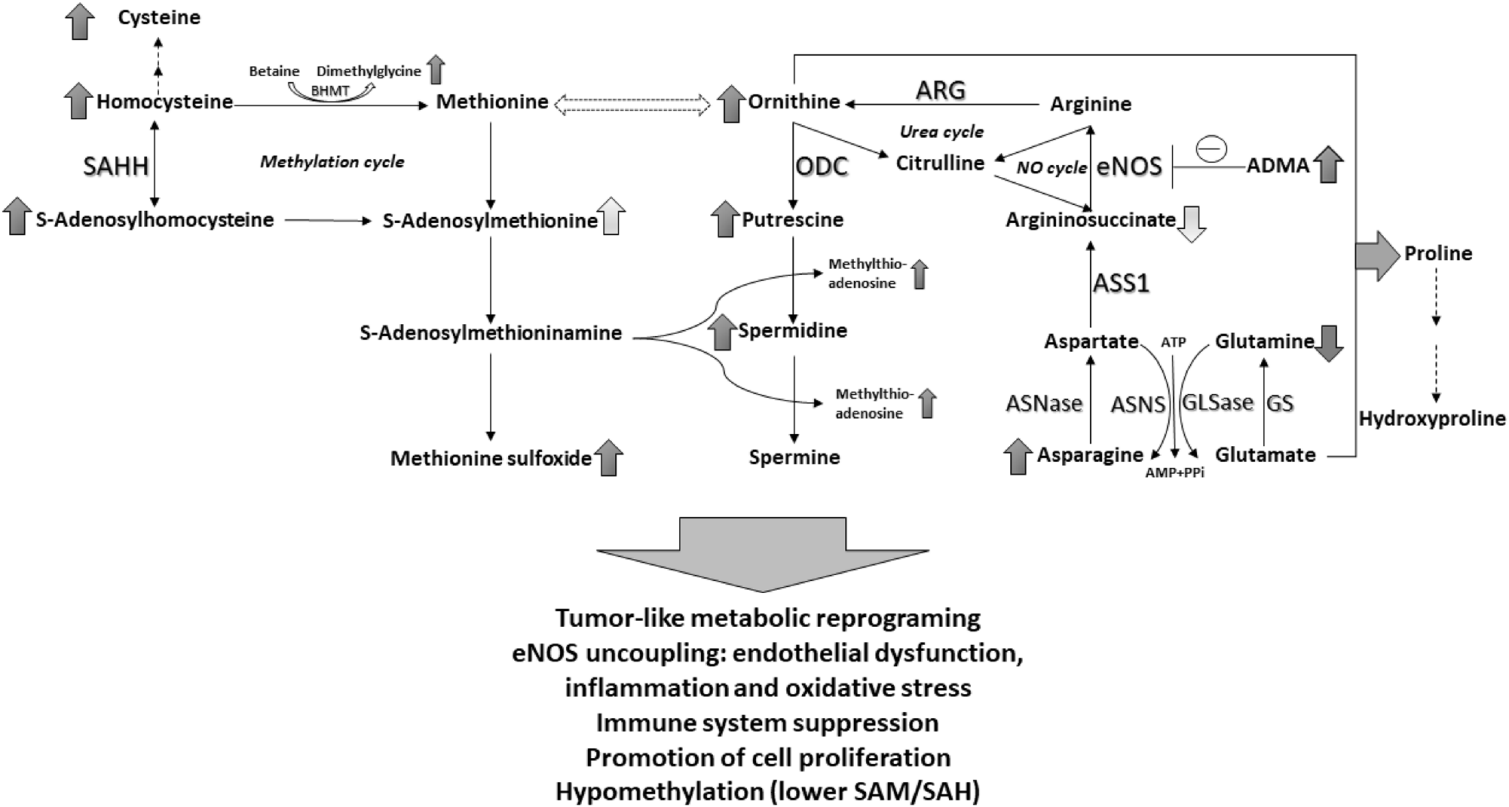
Summary of metabolic reprogramming observed in patients with ADPKD: changes in activity of NO and urea cycles, aspartate/asparagine and glutamine/glutamate cycles and methylation/ methionine cycles. Arrows indicate directional changes (increase/decrease) with lighter arrows expressing less pronounced changes. ADMA: asymmetric dimethylarginine, ARG: arginase1, ASNase: asparaginase, ASNS: asparagine synthetase, ASS1: argininosuccinate synthase 1, eNOS: endothelial nitric oxide synthase, GLSase: glutaminase, GS: glutamine synthetase, ODC: ornithine decarboxylase, SAM: S-adenosylmethionine, SAH: S-adenosylhomocysteine, SAHH: S-adenosylhomocysteine hydrolase.

Interestingly, the metabolite with the highest ROC AUC and potential for differentiating between the healthy and diseased children was asparagine that was more than threefold higher in the patients with ADPKD. A recent pediatric study showed that asparagine is significantly increased in pediatric cancer patients as compared to healthy children^39^. The same study identified a decrease in glutamine levels in diseased versus healthy patients^39^, an observation that we also made in ADPKD patients (Fig. 4).

In cancer cells, metabolic reprogramming results in an increased cell utilization of glutamine needed for sustaining the increased energy needs of rapidly dividing cells^40^. The expression of glutaminase, the enzyme that converts glutamine to glutamate in the mitochondria, is increased and glutaminase inhibitors are currently used in clinical trials as treatment options for diverse cancers^41,42^. Furthermore, asparagine synthetase that uses the amide group from glutamine to synthesize asparagine from aspartate, has also been shown to be overexpressed in cancers^43^. The opposite reaction of asparagine degradation is mediated by asparaginase, that is downregulated in cancer and, in its purified form, has been used for treatment of acute lymphoblastic (ALL) and acute myeloid leukemia (AML) for decades^44^.

In PKD, a recent study found that embryonic day 12.5 *Pkd1*-mutant but not wild-type kidneys require glutamine for growth^45^. In addition, treatment of *Pkd1*-mutant mice with a glutaminase inhibitor in utero (by administration to the mother) and postnatally (up to P10) slowed cyst progression^45^. Another study showed that glucose metabolism via TCA cycle was reduced and replaced by enhanced utilization of glutamine in *Pkd1*^−*/*−^ mouse embryonic fibroblasts^46^. It was found that these cells depend on glutamine for growth and show increased asparagine synthetase activity, the inhibition of which resulted in decreased cell growth^46^. Our results in patients support these recent studies (Fig. 4). Unfortunately, lisinopril and pravastatin were not able to alter these pathways, with plasma glutamine and glutamate concentrations further declining over time in the ACEI and ACEI plus statin-treated patients.

### Kynurenine and indole-related pathways

Plasma concentrations of kynurenate, a marker of renal insufficiency and oxidative stress^47,48^, was higher in ADPKD patients as compared to healthy subjects and further increased over time, independent of the treatment group. Interestingly, metabolomics analysis of plasma from 1434 adult participants of the Framingham Heart Study found kynurenic acid to be significantly increased in patients who progressed faster through CKD^49^. In the context of cystic kidney diseases, serum metabolomics analysis of adult participants of the Modification of Diet in Renal Disease Study showed higher levels of kynurenic acid in patients with polycystic kidney disease compared to patients with other causes of CKD^50^. Oppositely, another recent study showed no significant difference in the kynurenate levels between healthy subjects (n = 25) and ADPKD patients with preserved kidney function (eGFR ≥ 90 ml/min per 1.73 m^2^, n = 31) or between patients with ADPKD (n = 95) and CKD patients (n = 92) (eGFR < 90 ml/min per 1.73 m^2^ in both groups)^51^.

Indoleamine 2, 3-dioxygenases (IDO1 and IDO2) and tryptophan 2, 3-dioxygenase (TDO) are tryptophan catabolic enzymes that catalyze the conversion of tryptophan to kynurenine. The increase in kynurenine has been shown to exert important immunosuppressive functions by activating T-regulatory cells and myeloid-derived suppressor cells^52,53^. Targeting IDO1 represents a therapeutic opportunity in cancer immunotherapy and might be an effective strategy for targeting ADPKD as well.

Furthermore, we observed a reduction in plasma concentrations of aminoadipic acid in the placebo group over the 36-months observation period. Studies have shown aminoadipic acid inhibits the production of kynurenic acid in slices of kidney tissue, supporting the inverse relationship between these two metabolites in our results^54^.

Additionally, plasma levels of indole-3-carboxylic acid and indoleacetic acid (IAA) acid increased over time in both placebo and pravastatin groups, diverging further from healthy subjects. IAA has been linked to the oxidative stress and pro-inflammatory state seen in CKD and ADPKD^55^.

### Metabolites of disease progression

In addition to the above discussed accumulation of metabotoxins SAH and kynurenate, glycolysis intermediates 1,3-bisphosphoglycerate, 3-phosphoglycerate and 2-hydroxyglutarate (2-HG) also accumulated and were positively correlated with the percent change in HtTKV. Glucose metabolism is altered in ADPKD in a pattern similar to the Warburg effect found in cancer that produces a shift in energy production from mitochondrial oxidative phosphorylation to aerobic glycolysis^56–58^. 2-HG is an oncometabolite produced from α-ketoglutarate that causes genetic instability, affects T cell differentiation and immunity, and interacts with and modifies hypoxia and cell proliferation (mTOR) pathways^59,60^. Accumulation of 2-HG has been shown to occur in kidneys of B6(Cg)-*Cys1*^*cpk/*^J (*cpk*) mouse model of recessive PKD^61^.

1-methyladenosine and 3-methylhistidine are uremic solutes shown to accumulate in plasma and to be associate with renal disease^31,62^. Similarly, 4-pyridoxic acid and thus vitamin B6 metabolism have been shown to be altered in patients with renal diseases^63^, and seems to occur in early pediatric ADPKD as well.

In summary, our study is one of very few metabolomics studies in ADPKD patients, especially in children and young adults. We identified metabolites and metabolic pathways that are involved in the progression of ADPKD in pediatric patients. Said metabolites may allow for prognosis of ADPKD progression and stratification of patients into slow and rapid progressors to ESRD.

### Study limitations

Our study’s primary limitation was the lack of a disease control group that received no treatment (i.e. no ACE inhibitor therapy). RAAS activation is a prominent feature of ADPKD pathophysiology and ACE inhibitor therapy in children with ADPKD and borderline hypertension has been shown to prevent GFR decline and left ventricular mass index (LVMI) increase that otherwise occurs with disease progression. Given this, ACE inhibitor therapy was deemed standard of care and was provided to all patients in the clinical trial. Consequently, we were unable to definitively attribute metabolite changes to disease progression independent of ACE inhibitor treatment. Furthermore, no genotyping was performed in our patients and thus no adjustments could be made for either *PKD1* or *PKD2* mutations.

Furthermore, healthy subjects were on average 4 years younger than the ADPKD patients at baseline. Despite the adjustment for age as a confounding variable in our analyses, it should still be noted that some of the differences between the healthy and children with ADPDK might arise from the puberty-related metabolic changes.

Metabolomics studies of adults and children with ADPKD are limited, thus, when necessary, we compared our observed metabolite trends to those published for CKD patients.

A targeted, semi-quantitative assay with multiple quality control measures was used to reduce the false positive rate in detecting metabolites of importance. In the next step, specific targeted, validated assays for the herein identified candidate marker metabolites should be used in future larger clinical biomarker validation studies.

## Conclusions

The present study demonstrates that metabolites from the tryptophan, arginine, glutamine and asparagine metabolic pathways, urea and methylation cycles, are markedly associated with the development and progression of ADPKD. Statin therapy seemed to have limited effect on the levels of the metabolites monitored in the present study; it seemed that metabolite changes were primarily governed by ADPKD progression and/or ACE inhibitor treatment.

Currently, TKV is the only metric that has been recommended by the US FDA as a prognostic enrichment biomarker for the selection and inclusion of high risk ADPKD patients in clinical trials. The identification of plasma biomarkers, such as markers in the tryptophan metabolic pathway as well as in the urea and arginine cycles that can be used to monitor disease progression and treatment efficacy are potentially of great clinical utility. Further mechanistic qualification and prospective clinical studies are necessary to understand the changes in the described pathways and if these provide the basis for novel tools for the diagnosis and monitoring of human ADPKD progression.

## Supplementary Information

Supplementary Information.

## References

[1] Reddy BV, Chapman AB (2017). The spectrum of autosomal dominant polycystic kidney disease in children and adolescents. Pediatr. Nephrol..

[2] Torres VE, Harris PC, Pirson Y (2007). Autosomal dominant polycystic kidney disease. Lancet.

[3] Rahbari-Oskoui F, Williams O, Chapman A (2014). Mechanisms and management of hypertension in autosomal dominant polycystic kidney disease. Nephrol. Dial. Transpl..

[4] Ecder T, Schrier RW (2001). Hypertension in autosomal-dominant polycystic kidney disease: Early occurrence and unique aspects. J. Am. Soc. Nephrol..

[5] Chapman AB, Stepniakowski K, Rahbari-Oskoui F (2010). Hypertension in autosomal dominant polycystic kidney disease. Adv. Chronic Kidney Dis..

[6] Massella L (2018). Prevalence of hypertension in children with early-stage ADPKD. Clin. J. Am. Soc. Nephrol..

[7] Marlais M (2016). Hypertension in autosomal dominant polycystic kidney disease: A meta-analysis. Arch Dis Child.

[8] Augustyniak-Bartosik, H. *et al. The phenotypic characteristics of adult polycystic kidney disease have greater impact on the course of progressive disease than the type of mutation of the polycystin 1 gene*. Vol. 17 (2008).

[9] Johnson AM, Gabow PA (1997). Identification of patients with autosomal dominant polycystic kidney disease at highest risk for end-stage renal disease. J. Am. Soc. Nephrol..

[10] Schrier RW (2014). Predictors of autosomal dominant polycystic kidney disease progression. J. Am. Soc. Nephrol..

[11] Cadnapaphornchai MA (2011). Effect of statin therapy on disease progression in pediatric ADPKD: Design and baseline characteristics of participants. Contemp. Clin. Trials.

[12] Cadnapaphornchai MA (2014). Effect of pravastatin on total kidney volume, left ventricular mass index, and microalbuminuria in pediatric autosomal dominant polycystic kidney disease. Clin. J. Am. Soc. Nephrol..

[13] Peng B, Li H, Peng XX (2015). Functional metabolomics: From biomarker discovery to metabolome reprogramming. Protein Cell.

[14] Taylor SL (2010). A metabolomics approach using juvenile cystic mice to identify urinary biomarkers and altered pathways in polycystic kidney disease. Am. J. Physiol. Renal Physiol..

[15] Menezes LF, Lin CC, Zhou F, Germino GG (2016). Fatty acid oxidation is impaired in an orthologous mouse model of autosomal dominant polycystic kidney disease. EBioMedicine.

[16] Trott JF (2018). Arginine reprogramming in ADPKD results in arginine-dependent cystogenesis. Am. J. Physiol. Renal Physiol..

[17] Kim K, Trott JF, Gao G, Chapman A, Weiss RH (2019). Plasma metabolites and lipids associate with kidney function and kidney volume in hypertensive ADPKD patients early in the disease course. BMC Nephrol..

[18] Zacchia M (2019). Proteomics and metabolomics studies exploring the pathophysiology of renal dysfunction in autosomal dominant polycystic kidney disease and other ciliopathies. Nephrol. Dial. Transplant.

[19] Klawitter J (2015). Pravastatin therapy and biomarker changes in children and young adults with autosomal dominant polycystic kidney disease. Clin. J. Am. Soc. Nephrol..

[20] Davidson JA (2018). Metabolomic fingerprinting of infants undergoing cardiopulmonary bypass: Changes in metabolic pathways and association with mortality and cardiac intensive care unit length of stay. J. Am. Heart. Assoc..

[21] Yuan M, Breitkopf SB, Yang X, Asara JM (2012). A positive/negative ion-switching, targeted mass spectrometry-based metabolomics platform for bodily fluids, cells, and fresh and fixed tissue. Nat. Protoc..

[22] Chong J (2018). MetaboAnalyst 4.0: Towards more transparent and integrative metabolomics analysis. Nucleic Acids Res..

[23] den Bakker E, Gemke R, Bokenkamp A (2018). Endogenous markers for kidney function in children: A review. Crit. Rev. Clin. Lab. Sci..

[24] Work DF, Schwartz GJ (2008). Estimating and measuring glomerular filtration rate in children. Curr. Opin. Nephrol. Hypertens..

[25] Nowak KL, Cadnapaphornchai MA, Chonchol MB, Schrier RW, Gitomer B (2016). Long-term outcomes in patients with very-early onset autosomal dominant polycystic kidney disease. Am. J. Nephrol..

[26] Shamshirsaz AA (2005). Autosomal-dominant polycystic kidney disease in infancy and childhood: progression and outcome. Kidney Int..

[27] Toyohara T (2010). Metabolomic profiling of uremic solutes in CKD patients. Hypertens. Res..

[28] Boelaert J (2017). Metabolic profiling of human plasma and urine in chronic kidney disease by hydrophilic interaction liquid chromatography coupled with time-of-flight mass spectrometry: A pilot study. Anal. Bioanal. Chem..

[29] Duranton F (2014). Plasma and urinary amino acid metabolomic profiling in patients with different levels of kidney function. Clin. J. Am. Soc. Nephrol..

[30] Vanholder R (2003). Review on uremic toxins: Classification, concentration, and interindividual variability. Kidney Int..

[31] Mutsaers HA (2013). Optimized metabolomic approach to identify uremic solutes in plasma of stage 3–4 chronic kidney disease patients. PLoS ONE.

[32] Nigam SK, Bush KT (2019). Uraemic syndrome of chronic kidney disease: Altered remote sensing and signalling. Nat. Rev. Nephrol..

[33] Vanholder R, Pletinck A, Schepers E, Glorieux G (2018). Biochemical and clinical impact of organic uremic retention solutes: A comprehensive update. Toxins.

[34] Xia Y, Dawson VL, Dawson TM, Snyder SH, Zweier JL (1996). Nitric oxide synthase generates superoxide and nitric oxide in arginine-depleted cells leading to peroxynitrite-mediated cellular injury. Proc. Natl. Acad. Sci. USA.

[35] Pernow J, Jung C (2013). Arginase as a potential target in the treatment of cardiovascular disease: Reversal of arginine steal?. Cardiovasc. Res..

[36] Monticelli LA (2016). Arginase 1 is an innate lymphoid-cell-intrinsic metabolic checkpoint controlling type 2 inflammation. Nat. Immunol..

[37] Poillet-Perez L (2018). Autophagy maintains tumour growth through circulating arginine. Nature.

[38] Yang Y (2018). Interactions between macrophages and cyst-lining epithelial cells promote kidney cyst growth in pkd1-deficient mice. J. Am. Soc. Nephrol..

[39] Synakiewicz A (2017). Amino acid profiles as potential biomarkers for pediatric cancers: A preliminary communication. Biomark. Med..

[40] Metallo CM (2011). Reductive glutamine metabolism by IDH1 mediates lipogenesis under hypoxia. Nature.

[41] Mates JM (2019). Metabolic reprogramming of cancer by chemicals that target glutaminase isoenzymes. Curr. Med. Chem..

[42] Mates JM, Di Paola FJ, Campos-Sandoval JA, Mazurek S, Marquez J (2020). Therapeutic targeting of glutaminolysis as an essential strategy to combat cancer. Semin. Cell Dev. Biol..

[43] Lomelino CL, Andring JT, McKenna R, Kilberg MS (2017). Asparagine synthetase: Function, structure, and role in disease. J. Biol. Chem..

[44] Crowther D (1971). l-asparaginase and human malignant disease. Nature.

[45] Flowers EM (2018). Lkb1 deficiency confers glutamine dependency in polycystic kidney disease. Nat. Commun..

[46] Podrini C (2018). Dissection of metabolic reprogramming in polycystic kidney disease reveals coordinated rewiring of bioenergetic pathways. Commun. Biol..

[47] Pawlak K, Brzosko S, Mysliwiec M, Pawlak D (2009). Kynurenine, quinolinic acid–the new factors linked to carotid atherosclerosis in patients with end-stage renal disease. Atherosclerosis.

[48] Schefold JC (2009). Increased indoleamine 2,3-dioxygenase (IDO) activity and elevated serum levels of tryptophan catabolites in patients with chronic kidney disease: A possible link between chronic inflammation and uraemic symptoms. Nephrol. Dial. Transplant..

[49] Rhee EP (2013). A combined epidemiologic and metabolomic approach improves CKD prediction. J. Am. Soc. Nephrol..

[50] Grams ME (2017). Metabolomic alterations associated with cause of CKD. Clin. J. Am. Soc. Nephrol..

[51] Wang K (2020). Alterations of proximal tubular secretion in autosomal dominant polycystic kidney disease. Clin. J. Am. Soc. Nephrol..

[52] Liu M (2018). Targeting the IDO1 pathway in cancer: From bench to bedside. J. Hematol. Oncol..

[53] Platten M, Nollen EAA, Rohrig UF, Fallarino F, Opitz CA (2019). Tryptophan metabolism as a common therapeutic target in cancer, neurodegeneration and beyond. Nat. Rev. Drug Discov..

[54] Gramsbergen JBP (2002). Brain-specific modulation of kynurenic acid synthesis in the rat. J. Neurochem..

[55] Dou L (2015). The cardiovascular effect of the uremic solute indole-3 acetic acid. J. Am. Soc. Nephrol..

[56] Rowe I (2013). Defective glucose metabolism in polycystic kidney disease identifies a new therapeutic strategy. Nat. Med..

[57] Podrini C, Cassina L, Boletta A (2020). Metabolic reprogramming and the role of mitochondria in polycystic kidney disease. Cell Signal.

[58] Padovano V, Podrini C, Boletta A, Caplan MJ (2018). Metabolism and mitochondria in polycystic kidney disease research and therapy. Nat. Rev. Nephrol..

[59] Tyrakis PA (2016). S-2-hydroxyglutarate regulates CD8(+) T-lymphocyte fate. Nature.

[60] Ye D, Guan KL, Xiong Y (2018). Metabolism, activity, and targeting of D- and L-2-hydroxyglutarates. Trends Cancer.

[61] Hwang VJ (2015). The cpk model of recessive PKD shows glutamine dependence associated with the production of the oncometabolite 2-hydroxyglutarate. Am. J. Physiol. Renal Physiol..

[62] Silva RE (2018). Predictive metabolomic signatures of end-stage renal disease: A multivariate analysis of population-based data. Biochimie.

[63] Busch M (2010). Vitamin B6 metabolism in chronic kidney disease–relation to transsulfuration, advanced glycation and cardiovascular disease. Nephron Clin. Pract..

