# Supplementary material for: Metabolic profiling in children and young adults with autosomal dominant polycystic kidney disease
Source: Sci Rep. 2021 Mar 23;11:6629. doi: 10.1038/s41598-021-84609-8 (PMC7988179; doi:10.1038/s41598-021-84609-8)
Supplement: Supplementary file 1 — Supplementary Information. [file 41598_2021_84609_MOESM1_ESM.docx]

**SUPPLEMENTARY MATERIALS**

**METABOLIC PROFILING IN CHILDREN AND YOUNG ADULTS WITH AUTOSOMAL DOMINANT POLYCYSTIC KIDNEY DISEASE**

Madhurima M. Baliga^1^, Jost Klawitter^2^, Uwe Christians^2^, Katharina Hopp^3^, Michel Chonchol^3^, Berenice Y. Gitomer^3^, Melissa A. Cadnapaphornchai^3,4^, Jelena Klawitter^2,3^

^1^University of Colorado Denver, School of Medicine, Aurora, CO

^2^Deparment of Anesthesiology, University of Colorado Denver, Aurora, CO

^3^University of Colorado School of Medicine, Division of Renal Diseases and Hypertension, Aurora, CO

^4^Presbyterian/St. Luke’s Medical Center, Denver, CO

*Corresponding Author:*

Jelena Klawitter, PhD

Department of Anesthesiology

University of Colorado Denver Anschutz Medical Campus

Bioscience 2, Suite 200

12705 E Montview Blvd

Aurora, CO 80045-7109, USA

e-mail: [Jelena.Klawitter@cuanschutz.edu](mailto:Jelena.Klawitter@cuanschutz.edu)

**Methods**

***Trial Design***

Participants were randomized to pravastatin or placebo treatment in a double-blind manner with stratification by sex, age group, and hypertension status (hypertensive or normotensive).

***Measurement of Metabolites***

Sample analysis was performed using an Agilent 1200 series HPLC system (Agilent Technologies, Palo Alto, CA) interfaced with an ABSciex 5500 hybrid triple quadrupole/linear ion trap mass spectrometer (Concord, ON, Canada) equipped with an electrospray ionization source operating in the positive/ negative switch mode.

The Q1 (precursor ion) and Q3 (fragment ion) transitions, the metabolite names, dwell times and the appropriate collision energies for both positive and negative ion modes were adapted from Yuan et al. with several metabolite transitions added by our group. In total, Q1 and Q3 transitions were set to unit resolution for optimal metabolite ion isolation and selectivity. In addition, the polarity switching (settling) time was set to 50 ms. In 1.42 s using a 3-ms dwell time, we were able to obtain 6-14 scans per metabolite peak. Eight μL of sample was injected onto an Amide XBridge HPLC column (3.5 μm; 4.6 mm inner diameter [i.d.] × 100 mm length) (Waters, Milford, MA). The mobile phases consisted of HPLC buffer A (pH = 9.0: 95% (vol/vol) water, 5% (vol/vol) acetonitrile, 20 mM ammonium hydroxide, 20 mM ammonium acetate) and HPLC buffer B: 100% acetonitrile. The HPLC settings were as follows: from 0 to 3 minutes, the mobile phase was kept at 85% B; from 3 to 22 minutes, the percentage of solvent B was decreased from 85% to 2% and was kept at 2% for additional 3 minutes. At minute 26, solvent B was increased again back to 85% and the column flushed for additional 7 minutes at 85% solvent B.

**SUPPLEMENTARY TABLES**

**Supplementary Table Legends**

**Supplementary Table 1: Significantly changed metabolites in placebo and pravastatin groups over time.** Within the placebo and pravastatin groups, analysis was performed for patients with data for both baseline and 36-month time points. Metabolites that were significant after the Bonferroni correction and with an absolute log2(fold change) of >1.2 are presented in the table.

Abbreviations: 5-HIAA, 5-hydroxyindoleacetic acid; ADMA, asymmetric dimethylarginine; MMA, monomethylarginine; SAH, s-adenosylhomocysteine; SAM, s-adenosylmethionine; SDMA, symmetric dimethylarginine.

**Table 1:**

| Placebo | | | | Pravastatin | | | |
| --- | --- | --- | --- | --- | --- | --- | --- |
|  | Fold Change (FC) | log2(FC) | P |  | Fold Change (FC) | log2(FC) | P |
| 5-HIAA | 0.36 | -1.46 | <0.001 | 5-HIAA | 0.34 | -1.56 | <0.001 |
| adenine | 0.31 | -1.69 | <0.001 | adenine | 0.34 | -1.55 | <0.001 |
| argininosuccinate | 0.26 | -1.96 | <0.001 | argininosuccinate | 0.13 | -2.89 | <0.001 |
| ascorbic acid | 0.26 | -1.94 | <0.001 | ascorbic acid | 0.26 | -1.95 | <0.001 |
| atrolactic acid | 0.21 | -2.27 | <0.001 | atrolactic acid | 0.20 | -2.32 | <0.001 |
| carbamoyl phosphate | 0.37 | -1.44 | <0.001 | carbamoyl phosphate | 0.19 | -2.37 | <0.001 |
| cystathionine | 0.36 | -1.48 | <0.001 | cystathionine | 0.29 | -1.78 | <0.001 |
| cysteine | 0.20 | -2.29 | <0.001 | cysteine | 0.18 | -2.48 | <0.001 |
| cystine | 0.27 | -1.91 | <0.001 | cystine | 0.22 | -2.22 | <0.001 |
| indole | 0.32 | -1.65 | <0.001 | indole | 0.31 | -1.71 | <0.001 |
| indole-3-carboxylic acid | 4.21 | 2.07 | <0.001 | indole-3-carboxylic acid | 4.11 | 2.04 | <0.001 |
| indoleacrylic acid | 3.30 | 1.72 | <0.001 | indoleacrylic acid | 3.08 | 1.62 | <0.001 |
| lysine | 0.40 | -1.33 | <0.001 | lysine | 0.43 | -1.21 | <0.001 |
| niacin | 0.31 | -1.67 | <0.001 | niacin | 0.37 | -1.45 | <0.001 |
| ornithine | 0.41 | -1.29 | <0.001 | ornithine | 0.43 | -1.22 | <0.001 |
| pipecolic acid | 0.29 | -1.77 | <0.001 | pipecolic acid | 0.23 | -2.12 | <0.001 |
| proline | 0.32 | -1.64 | <0.001 | proline | 0.41 | -1.30 | <0.001 |
| purine | 0.42 | -1.25 | <0.001 | purine | 0.40 | -1.34 | <0.001 |
| SAM | 0.22 | -2.16 | <0.001 | SAM | 0.04 | -4.63 | <0.001 |
| tyrosine | 0.40 | -1.33 | <0.001 | tyrosine | 0.43 | -1.23 | <0.001 |
| uracil | 0.35 | -1.51 | <0.001 | uracil | 0.34 | -1.55 | <0.001 |
| uridine | 0.28 | -1.86 | <0.001 | uridine | 0.32 | -1.63 | <0.001 |
| xanthurenic acid | 0.39 | -1.37 | <0.001 | xanthurenic acid | 0.16 | -2.68 | <0.001 |
| methylcysteine | 0.32 | -1.66 | <0.001 | 2-isopropylmalic acid | 0.41 | -1.30 | <0.001 |
| phenyllactic acid | 2.36 | 1.24 | <0.001 | glucosamine | 0.35 | -1.52 | <0.001 |
|  |  |  |  | glutamate | 0.43 | -1.21 | <0.001 |
|  |  |  |  | glycerate | 0.43 | -1.22 | <0.001 |
|  |  |  |  | hydroxyproline | 0.29 | -1.79 | <0.001 |
|  |  |  |  | methylguanosine | 0.41 | -1.28 | <0.001 |
|  |  |  |  | N-acetylputrescine | 0.42 | -1.25 | <0.001 |
|  |  |  |  | p-hydroxybenzoate | 0.40 | -1.32 | <0.001 |
|  |  |  |  | SAH | 0.32 | -1.63 | <0.001 |
|  |  |  |  | SDMA | 0.36 | -1.49 | <0.001 |

**SUPPLEMENTARY** **FIGURES**

**Supplementary Figure 1:**

**Effect of pravastatin on the progression of ADPKD.** Percent changes in HtTKV between 36 months and 0 months was calculated for the pravastatin group (N=31) and compared to the placebo group (N=27).

P = 0.044

**Supplementary Figure 2:**

**(A) Principal component analysis scores plot of children with ADPKD at baseline (before the initiation of pravastatin treatment (A0) versus placebo group (B0), respectively).** Ellipses represent 95 % confidence intervals for each individual group on the PCA plot.

**
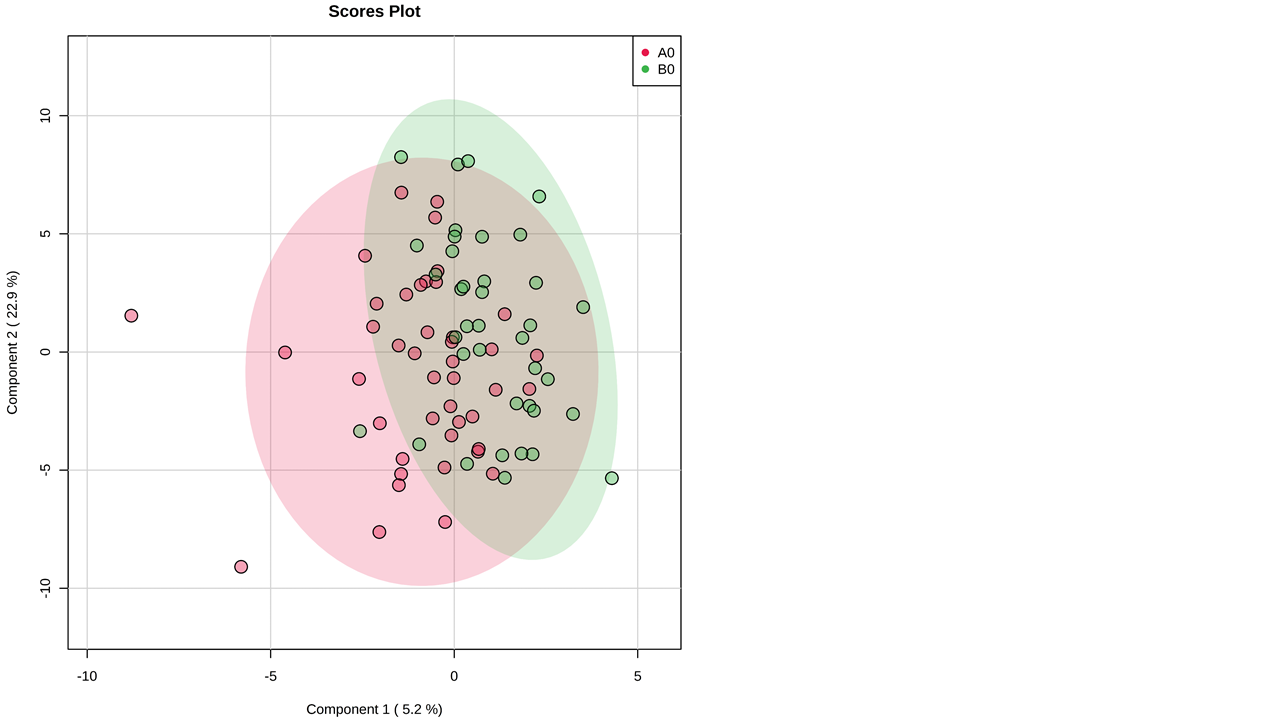
**
